# Bacterial cellulose scaffolds derived from brewing waste for cultivated meat applications

**DOI:** 10.3389/fnut.2025.1656960

**Published:** 2025-09-19

**Authors:** Christian Harrison, Elif Gokoglan, Richard M. Day

**Affiliations:** Day Lab, Department of Aging, Rheumatology and Regenerative Medicine, UCL Division of Medicine, University College London, London, United Kingdom

**Keywords:** cultivated meat, cellular agriculture, alternative protein, 3D scaffold, bacterial cellulose, tissue engineering, cultivated meat production

## Abstract

**Introduction:**

The negative externalities of conventional meat production are driving a search for sustainable alternative proteins. Cultivated meat (CM) is one such alternative, but its development is constrained by the need for sustainable, edible, and low-cost cellular scaffolds that can replicate animal tissue texture and structure. Bacterial cellulose (BC) derived from brewer's spent yeast (BSY) could provide a scalable and affordable scaffold material for structured CM products.

**Methods:**

The composition of BSY and its ability to support BC growth were assessed through metabolic analysis and growth trials. Properties relevant to CM applications of BSY-derived BC were then investigated. Scanning electron microscopy was used to quantify surface porosity. Mechanical properties were measured using texture profile analysis. Thermal and chemical properties were assessed using differential scanning calorimetry and Fourier-transform infrared spectroscopy, and biocompatibility was evaluated through cell attachment assays.

**Results:**

BSY supported BC production, yielding material with structural, thermal, and textural properties comparable to BC grown on conventional media and similar to conventional meat products. BSY-derived BC also supported L929 fibroblasts, with 35.9% ± 2.5% cell attachment after 24 h and evidence of continued proliferation.

**Discussion:**

These findings demonstrate that BSY can be effectively valorized to produce BC scaffolds for CM. This approach offers a cost-effective and sustainable strategy to improve the scalability of cultivated meat, contributing to future sustainable food production.

## Introduction

Significant concerns have been raised over the negative externalities of current animal agriculture practices, particularly factory farming, for the environment, animal welfare and human health (1–3). Nonetheless, global demand for meat products is forecast to rise substantially in future decades (4). In developed countries meat consumption is mostly static, while in developing countries it is growing fast (5). A variety of solutions to meet global protein demand sustainably have been proposed, including those aiming for more sustainable agricultural practices (6). Among these are alternative proteins, of which there are several types (7). For some applications, alternative proteins aim to satisfy protein needs while reducing or replacing conventionally produced animal products in supply chains. For other applications, the aim is to offer multiple sources of proteins to consumers with no competition between them. One form of alternative proteins, plant-based meat analogs, have had some success but may have plateaued in popularity due to their high cost and inability to replicate the taste and texture of meat (8, 9). Another subset of alternative protein food products, cultivated meat (CM), seeks to produce meat products by culturing animal cells in controlled environments, without using any animal-derived ingredients. This approach may be better able to deliver products that meet consumer expectations. CM also has potential to be a more sustainable alternative to conventional meat production through lower land use, water consumption and greenhouse gas emissions, and potential for more ethical and efficient food production (9). However, these benefits are currently uncertain, due to the difficulty of conducting life cycle assessments at this early stage of development and will depend heavily on the exact materials and energy sources used for CM production (10). Additionally, a number of significant technical hurdles remain before CM can become a viable process capable of reaching a mass market.

If they are to displace conventional meat products, alternative proteins must be competitive on price, texture, taste, nutrition, and convenience as well as safe. These factors are crucial for any potential consumer and regulatory acceptance of CM products (11, 12). Achieving parity with conventional meat has been challenging and alternative proteins have so far failed to gain widespread acceptance (7). Part of the problem is the difficulty of replicating the complex hierarchical structure of animal tissues that produces the taste and texture of meat (13). This cannot be achieved using cells alone, which are conventionally grown in 2D formats and further constrained by using non-edible substrate materials. Edible tissue scaffolds could solve this by providing a 3D framework to support and guide cell growth and differentiation and contributing additional desirable organoleptic properties (13). To be suitable for mass production, scaffold materials must be cheap, biocompatible, and safe for consumption, ideally porous to facilitate cell ingression and nutrient perfusion, and malleable to allow production of varied and aesthetically pleasing products. A variety of materials have been investigated as potential scaffolds for tissue engineering related to healthcare applications, such as synthetic polymers and decellularized plant material (13). However, many of these scaffolds are too expensive for mass food production at a competitive price point, inappropriate for human consumption or insufficiently biodegradable for human digestion.

Bacterial cellulose (BC) is an abundant alternative material, a biopolymer of glucose synthesized by several bacterial species, such as *Komagataeibacter xylinus*, for purposes of mechanical, chemical and biological protection in their environment (14). BC consists of a polysaccharide of glucose units linked by β(1 → 4) bonds, with intrachain hydrogen bonds that add strength to the structure (15). It is chemically identical to plant cellulose, but lacks the lignin, hemicellulose and pectin found in plants that necessitate chemical purification for CM applications which adds expense and may not be food-safe (16). In static cultures, BC is produced in the form of a pellicle covering the surface of the growth medium, while in agitated cultures BC may form in balls or strands (14). Several properties make it well-suited as a scaffold material for cell attachment and growth, including its web-like network structure, tensile strength, and high water-holding capacity (17). BC is structurally similar to the extracellular matrix and if processed correctly, has high purity and low cytotoxicity (18, 19). Its relatively high porosity also enables ingress of cells into the material and facilitates diffusion of essential nutrients and waste products through the scaffold (18). Consequently, BC has been shown to be at least as biocompatible as other scaffold materials for various biomedical applications, as well as more affordable (20). Furthermore, BC pellicles can be formed in almost any desired shape (21), allowing it to masquerade as conventional meat products. Due to these properties, BC has been suggested as a scaffold material for CM (22). Studies have succeeded in culturing both human and animal myoblasts on BC, showing promise for CM applications, though further optimisation of cell attachment may be needed.

BC production requires culture medium containing a carbon source, such as glucose, a nitrogen source, such as peptone or yeast extract, and other critical components such as phosphorus and magnesium (23). Hestrin-Schramm (HS) medium is one of the most widely used formulations (24). 105 media is another similar formulation recommended for cultivation of BC producing bacteria such as *K. xylinus* (25). The cost of conventional culture medium such as HS may be a large component of total production costs and is one obstacle to potential use of BC in CM (26). However, BC can be produced using a wide range of alternative carbon and nitrogen sources. For example, the main commercial edible BC product currently is the dessert “nata de coco”, made using coconut water (27). Growth media derived from food or agricultural waste products are of particular interest; they are cheap, and using waste products avoids competition for inputs with other industries (28). If production methods using waste streams can be developed, these materials can be valorised, while also enabling BC to be made cheaply, at scale and more sustainably. No study thus far is known to have proposed using BC derived from industrial or agricultural wastes for CM production.

One such waste stream is brewing waste produced in the beer-making process. There are several types of brewing waste; brewers spent grain (BSG), trub, and brewers spent yeast (BSY) (29). All three have great potential as sources of carbohydrates, proteins and micronutrients for edible applications (30–32). Global beer production stands at ~1.9 billion hectolitres annually, and per hL of beer, around 20 kg of BSG and 0.3 kg of BSY are generated, resulting in large volumes of waste (33). Some brewing waste is repurposed as animal feed or as food additives to enhance taste or nutrition (29, 32). Despite this, brewing waste is underutilized and much of it is disposed in landfill, which can lead to water pollution or greenhouse gas release (34). Even if managed correctly disposal can impose significant costs on brewers (29). Incorporating brewing wastes into the CM supply chain would valorise this waste product, simultaneously reducing costs for brewers, and providing a sustainable feedstock for food production. Brewing wastes have already been trialed as a growth medium for BC-producing bacteria (35). We propose using brewing waste to grow BC, which can in turn act as a cellular scaffold for CM production. In this study we aim to demonstrate proof of concept for this production process.

## Materials and methods

### Sourcing of brewing waste

Brewers spent yeast (BSY) was collected from Big Smoke Brewing Company (Esher, UK). BSY was extracted from the bottom of the fermenter tank after primary fermentation and collected in 2 L borosilicate glass bottles. The brewing yeast used was strain W3470 of *Saccharomyces pastorianus*. Four batches of brewing waste were collected as required at intervals of approximately 2 months. As BSY was sourced directly from an active brewing process, the bulk ingredients used in the fermentation process were dependent on the brewer's production schedule at the time of collection and may have varied. After collection, BSY was autoclaved at 134 °C, then stored in 2 L borosilicate glass bottles at 4 °C for up to 6 months. Aliquots of BSY were taken under sterile conditions in a laminar flow hood as required for BC cultivation.

### Metabolic analysis of brewing waste

15 ml samples were taken from 4 different batches of BSY and centrifuged at 4,500 rcf for 10 min at room temperature. The resulting supernatant was collected and sent to Nova Biomedical (Runcorn, UK) for measurement of 8 common metabolites: glutamine, glutamate, glucose, lactate, ammonium (${\text{NH}}_{4}^{+}$), sodium (Na^+^), potassium (K^+^), and calcium (Ca^2+^). Samples were analyzed using a BioProfile FLEX2 Automated Cell Culture Analyzer which measured the concentration of 8 common metabolites. Samples were diluted in proprietary diluent as required to fall within the measurement range of the FLEX2.

### Bacterial cultivation

Freeze-dried *Komagetaeibacter xylinus* bacteria (DSM no. 2004) were sourced from DSMZ—German Collection of Microorganisms and Cell Cultures (Braunschweig, Germany). Optimal culture conditions for *K. xylinus* were followed according to the DSMZ database (105 media, 28 °C). 105 media was prepared according to the DSMZ formula, (without CaCO_3_). Media was autoclaved at 134 °C and subsequently stored at 4 °C.

*K. xylinus* bacteria were grown in static culture in standard T75 (surface area 75 cm^2^) tissue culture flasks filled with 50 ml 105 media. Once a pellicle was visible, liquid was collected from the culture and used to inoculate new cultures under sterile conditions in a laminar flow hood.

### Comparison of bacterial cellulose growth in different media

T25 cell culture flasks (surface area 25 cm^2^) were filled with 15 ml of either 105 media or BSY. Each flask was then inoculated with bacteria from a stock culture. O.D 600 nm of the bacterial stock used for inoculation was adjusted to ~0.1 at 600 nm before inoculation using an Eppendorf BioPhotometer, to ensure an equivalent starting concentration of bacteria between samples.

Bacterial cultures were grown in an incubator at 28 °C with humidified atmosphere. After 2 weeks the resulting BC pellicles were harvested and washed several times by stirring in dH_2_O at 400 rpm using a magnetic stirrer to remove as much of the growth medium as possible. Pellicles were frozen and freeze-dried using a Thermo Fisher MicroModulyo 1.5 L freeze-drier, until they reached a constant weight. To remove bacteria from the pellicles, samples were washed in 0.3 M NaOH at 80°C for 30 mins at 400 rpm using a magnetic stirrer hotplate. Pellicles were washed repeatedly in dH_2_O until the pH of the water returned to 7.0, then freeze-dried and weighed a second time. Cellulose yield after removal of bacteria was calculated in grams/liter of starting culture medium.

### Production of bacterial cellulose square pellicles

To test cell growth on BC, small uniform squares of BC were produced. Each well measuring 19 x 19 mm in a 25 well square non-tissue culture treated polystyrene petri dish was filled with 2 ml of either 105 media or BSY. Each well was then inoculated with 50 μl bacteria from a stock culture. Plates were then placed into a humidified incubator at 28 °C and left to grow for 14 days. This produced square cellulose pellicles of uniform thickness (~1 mm) with a surface area of ~3.7 cm^2^ each. Pellicles were harvested using forceps, rinsed in dH_2_O, then washed in 0.3 M NaOH at 80°C for 30 min using a magnetic stirrer at 400 rpm. Pellicles were then washed repeatedly in dH_2_O until the pH of the water returned to 7. Pellicles were then placed into bottles, immersed in dH_2_O and autoclaved at 134 °C. Pellicles were stored at room temperature until use or freeze-dried where specified for longer-term storage.

### Ultrastructural analysis

Square cellulose pellicles grown in 105 media and in various batches of brewing waste were freeze dried using a Thermo Fisher MicroModulyo 1.5 L freeze-drier. Pellicles were then glued to stubs using epoxy resin and sputter coated with a thin layer of gold-palladium (Au/Pd), using a Polaron E5000 sputter coater (Quorum, UK). SEM images were then acquired using a Sigma 300 VP Scanning Electron Microscope (Zeiss). Images were acquired at 200, 500, 1,000, 5,000 and 10,000 × magnification using an electron high tension of 5.00 kV. Several images were acquired from different fields of view for each cellulose pellicle.

### Surface porosity calculation

SEM images were analyzed using Fiji. For surface analysis of porosity, only images at 5,000 × magnification were used. Manual thresholding was used to identify pores, accounting for differences in contrast and brightness between images. Pores were defined as dark areas with visible pore boundaries, and the same thresholding criteria were used across all images. Percentage porosity was calculated as the area occupied by pores/total image area. A mean porosity value for each pellicle was calculated using 3 images taken from different areas of the pellicle surface. These mean values were then used in statistical analysis to identify any differences between treatments.

### Differential scanning calorimetry

Differential Scanning Calorimetry (DSC) analysis was performed on freeze-dried bacterial cellulose squares using a DSC25 pressure differential scanning calorimeter (TA Instruments, USA). 2–3 mg samples grown in both 105 medium and BSY were sealed in aluminum pans (Tzero Aluminum Hermetic) and heated from 25 °C to 300 °C at a rate of 10 °C/min under inert nitrogen gas (*N*_2_) at a flow rate of 50 mL/min.

### Fourier-transform infrared spectroscopy

Attenuated Total Reflectance Fourier-Transform Infrared (ATR-FTIR) spectroscopy was performed on the freeze-dried bacterial cellulose films using a Spectrum ONE FTIR spectrometer (PerkinElmer) equipped with an UATR accessory. Spectra were collected over the range of 4,000–500 cm^−1^, with 20 scans per sample at a resolution of 4 cm^−1^. For each sample, the background spectrum was recorded using an empty cell.

### Production of stacked cellulose-gel composites

Cellulose squares were first briefly immersed in a 3.25% (w/v) solution of culinary vegan setting agent Dr. Oetker VEGE-GEL, which primarily consists of dextrose and gelling agents (carrageenan and locust bean gum). Using forceps, a stack of 5 gel-soaked squares was assembled inside each well of a custom-made 3D-printed mold with internal dimensions matching the 100 mm square non-tissue culture treated polystyrene petri dishes. Each well was a 3.7 cm^2^ square and the mold contained a removable base. Additional gel was poured over the stack to fill any air gaps in each well before the mold was inverted and pressed down, leaving it resting face-down and flush with a flat surface. After leaving the gel to set for 12 h at room temperature, the base of the mold was removed, revealing ~5 mm thick cubes of gel-encased cellulose. Cubes were stored in a sealed petri dish at 4 °C for up to 1 week before use.

### Texture profile analysis of stacked cellulose-gel composites

Stacked cellulose composites were sent to Stable Micro Systems (Surrey, UK) for texture profile analysis (TPA). A TA.XTplusC texture analyser with 40 mm cylinder probe was used to perform a double compression of the sample, simulating a biting action, as per a simplified TPA program. This provided data on the hardness, cohesiveness, springiness and adhesiveness of the sample. Further parameters including gumminess and chewiness were derived from these values.

### Cell seeding on cellulose pellicles

Cellulose squares grown in BSY only were transferred to sterile 100 mm 25 well square non-tissue culture treated polystyrene petri dishes. A single batch of mouse fibroblast L929 cells (Merck, UK) were thawed and expanded in flasks using serum-free medium [Gibco Dulbecco's Modified Eagle Medium/Nutrient mixture F-12 (ThermoFisher Scientific, UK) + 10% Proliferum^®^ B animal component-free serum alternative (Multus Biotechnology, UK)]. Cells were detached using trypsin-EDTA, pooled and counted using the Cell Count and Viability Assay function of the Chemometec Nucleoview NC200 (Chemometec, Denmark). Cells were seeded onto the cellulose squares at a concentration of 200,000 cells/well. Standard 12-well tissue culture plates were also seeded at the same concentration of cells for use as a control. 1 ml serum-free medium was added to each well. Plates were then incubated at 37 °C, 5% CO_2_ in a humidified atmosphere.

To determine cell attachment efficiency and proliferation at 24 and 72 h, cellulose squares from three wells in each group at each timepoint were removed. Cellulose squares were transferred using forceps into a fresh 100 mm 25 well square non-tissue culture treated polystyrene petri dish, then gently washed once with 1 ml sterile PBS to remove any unattached cells from counting. Similarly, cells from three wells at each time-point in the 12-well plates were gently washed once with 1 ml PBS. PBS was removed from all wells followed by addition of 200 μl Reagent A100 lysis buffer (Chemometec, Denmark) was applied to each well. After 5 min of incubation in lysis buffer at room temperature, 200 μl of Reagent B stabilizing buffer (Chemometec, Denmark), was added to each well. The total number of cells in each sample at each timepoint were counted using the count of Aggregated Cells A100 and B Assay function of the Nucleoview NC200.

### Fluorescence staining and cell imaging

Cells grown on cellulose and standard tissue culture plates were fixed prior to fluorescent fluorescent imaging of cells. Cellulose squares were first transferred with forceps to a fresh 25 well square non-tissue culture treated polystyrene petri dish, then both cellulose and control samples were gently washed 3 times with 1 ml PBS. Following this, 0.5 ml 10% formalin was applied to each well and incubated at room temperature for 5 min. The formalin was then removed, and samples washed another 3 times with 1 ml PBS. Samples were then stored in PBS at 4 °C for no more than 2 months until required. DAPI and phalloidin were used to stain cell nuclei and filamentous actin, respectively. Samples were first permeabilised with 0.5 ml of 0.1% Triton X-100 in PBS for 15 min. Samples were then washed twice with 1 ml PBS. A staining solution of 5 μl Invitrogen Alexa Fluor 568 phalloidin dye (Life Technologies Limited, ThermoFisher Scientific) in 200 μl PBS with 1% bovine serum albumin was prepared per sample. Samples were covered and incubated in the staining solution for 1 h at room temperature, after which the staining solution was removed. A second staining solution of DAPI was prepared, adding 2 drops of Nucblue Fixed Cell ReadyProbes Reagent (Thermo Fisher) per ml of PBS. 200 μl of this second solution was then added to each sample. Samples were covered and incubated for a further 5 min at room temperature. Samples were then washed twice more with 1 ml PBS. Stained cellulose samples were then placed face down on a glass microscope slide using forceps. Control samples were imaged directly in their wells. Fluorescence microscopy images were acquired using a DMi8 inverted fluorescence microscope (Leica).

### Figure generation

Figures were produced using the pandas v2.0.3, matplotlib v3.8.2, seaborn v0.13.2 and statannotations v0.7.2 Python packages and OriginPro2021.

## Statistical analysis

All statistical analysis was performed in Python using the pandas v2.0.3, scipy v1.11.1 and statsmodels v0.14.0, packages. All data were first assessed for normality and homogeneity of variances. Normality of residuals from a one-way ANOVA was evaluated using the Shapiro-Wilk test, and homogeneity of variances across groups tested using Levene's test.

For normally distributed data, Welch's *t* test was used for pairwise comparison.

Where data did not meet assumptions of normality and/or equal variance, non-parametric tests were used. When comparing multiple groups, the Kruskal-Wallis test was used to detect significant differences, followed if needed by pairwise Mann-Whitney *U* tests to identify specific group differences. When comparing only two groups, the Mann-Whitney U test was used to identify pairwise differences.

A significance threshold of *p* = 0.05 was applied throughout. Significant differences are indicated in plots using the following annotations: *p* < 0.05(^*^), < 0.01(^**^).

## Results

**Figure 1:**
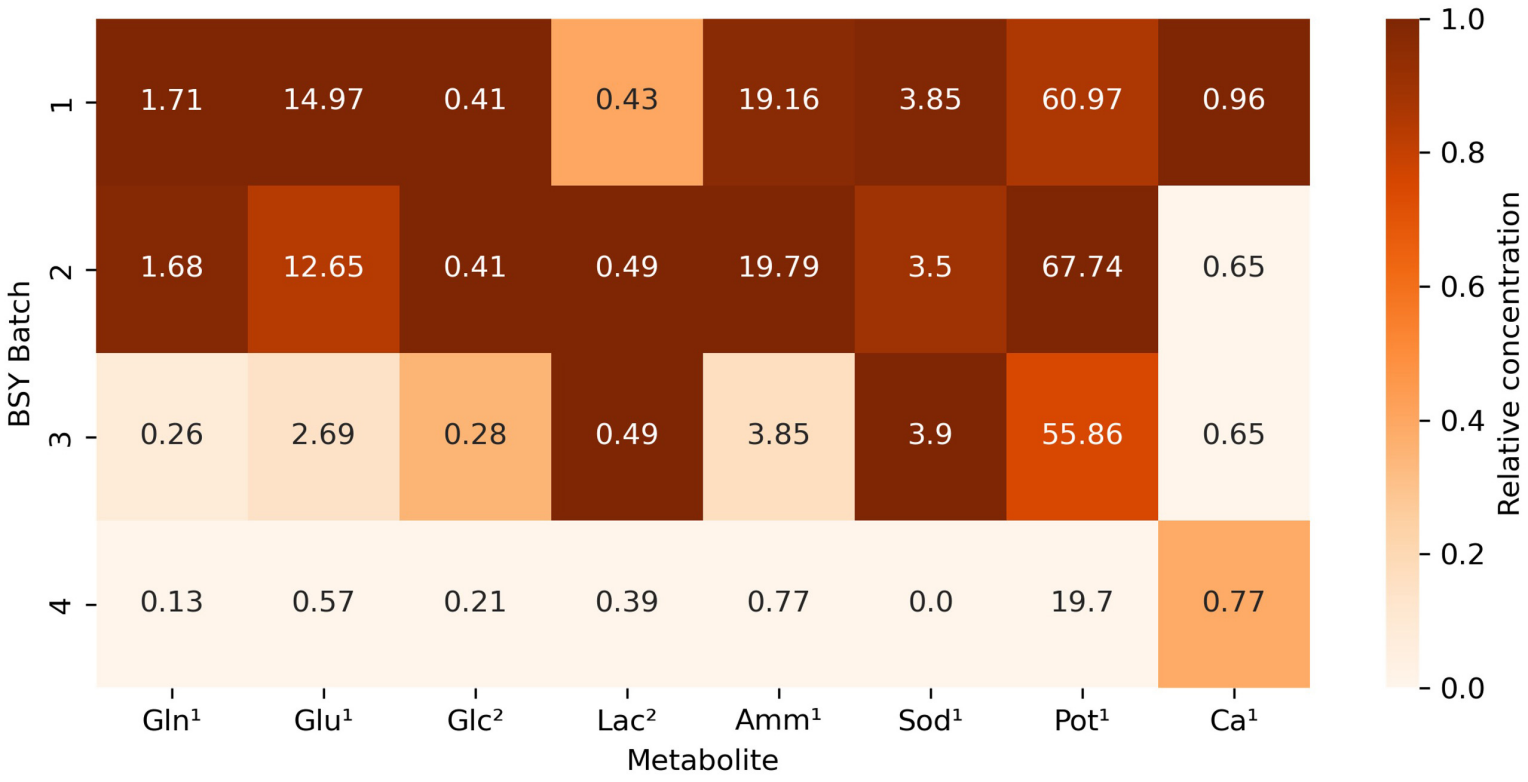
Heatmap showing relative concentrations of selected metabolites across various batches of BSY. Values refer to the measured value of each metabolite in each batch. Color intensity represents relative concentration of each metabolite between batches, with darker shades indicating higher concentrations. Metabolites are represented by short codes: Gln, glutamine; Glu, glutamate; Glc, glucose; Lac, lactate; Amm, ammonium; Sod, sodium, Pot, potassium; Ca, calcium. Superscript numbers denote units of measurement: ^1^mmol/L, ^2^g/L.

**Figure 2:**
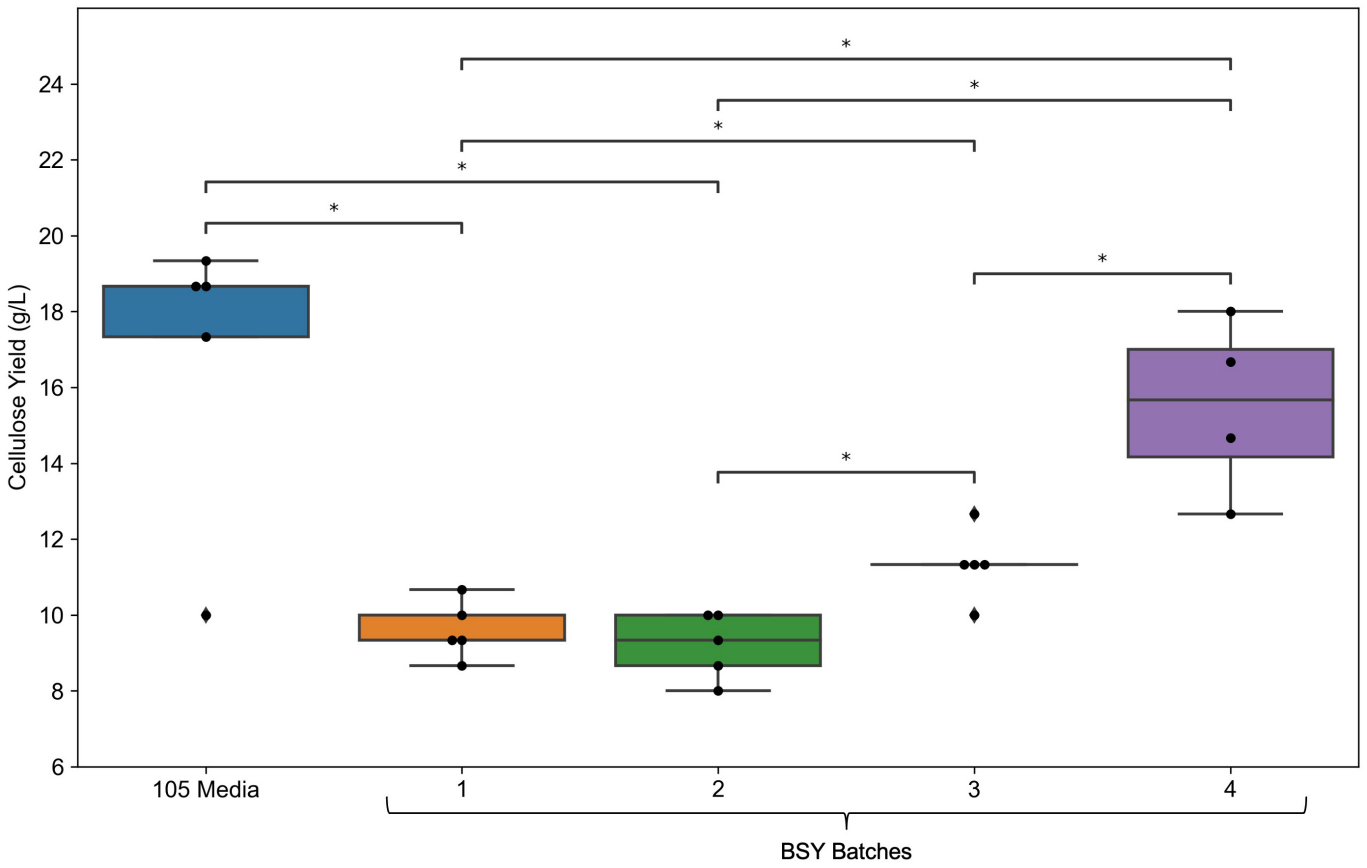
Comparison of bacterial cellulose yield between standard 105 media and various batches of BSY (*n* = 5). Dots represent individual data points, ♦ points represent outliers. Statistical comparisons were made using a Kruskal–Wallis test followed by Mann–Whitney *U* pairwise comparisons. Asterisks indicate significant pairwise differences: *p < 0.05.

Measurement of selected metabolites across different batches of BSY (Figure 1) showed substantial variation in levels of certain metabolites between batches. Variation in glutamine, ammonium (${\text{NH}}_{4}^{+}$), sodium (Na^+^) and potassium (K^+^) levels were particularly pronounced, with ammonium levels over 25 times higher in batch 1 than in batch 4. BC yield (Figure 2) varied across BSY batches, with bacteria grown in batches 3 and 4 producing significantly more cellulose than in batches 1 or 2. Notably, BC yields in batches 3 and 4 were not significantly different from that in standard 105 medium.

Of the 8 metabolites measured, 6 showed no significant correlation with BC yield (Table 1) Ammonium and potassium however were both strongly negatively correlated with BC yield.

**Table 1:** Spearman's rank correlation between metabolite concentrations and bacterial cellulose yield across different batches of BSY.

**Metabolite**	**Spearman ρ**	***p*-value**
Glutamine	−0.80	0.200
Glutamate	−0.80	0.200
Glucose	−0.95	0.051
Lactate	−0.63	0.368
${\text{NH}}_{4}^{+}$	−1.00	0.000
Na^+^	−0.20	0.800
K^+^	−1.00	0.000
Ca^2+^	0.21	0.789

Correlation coefficients (ρ) and corresponding *p*-values are reported for each metabolite. Statistical analysis was performed using scipy.stats.spearmanr in Python.

**Figure 3:**
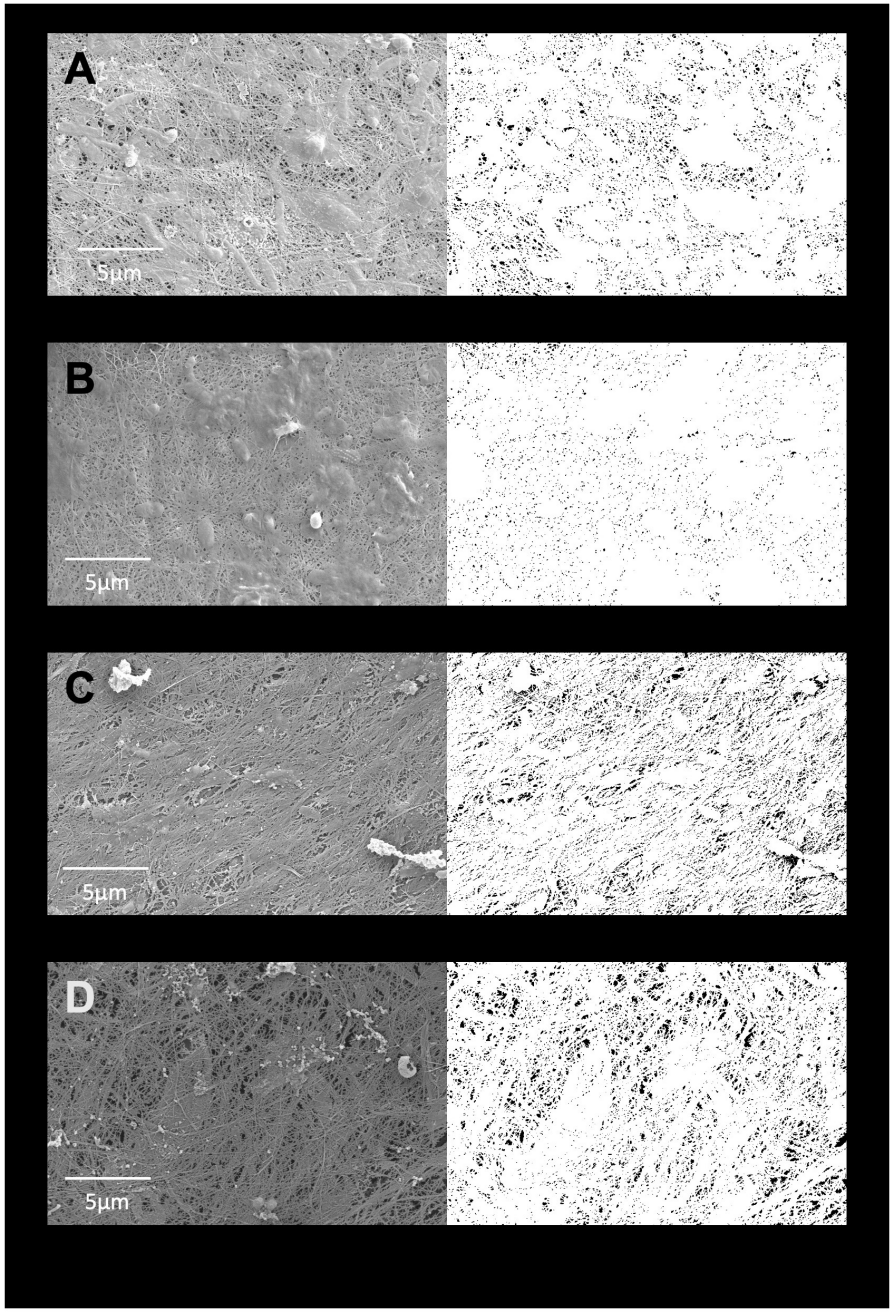
SEM images of cellulose pellicles grown in either standard media **(A)**, BSY batch 2 **(B)**, BSY batch 3 **(C)** or BSY batch 4 **(D)**. To the left are the original images, to the right are the corresponding binary masks generated by manual thresholding used to calculate % porosity. Scale bars are provided at the bottom of each original image.

**Figure 4:**
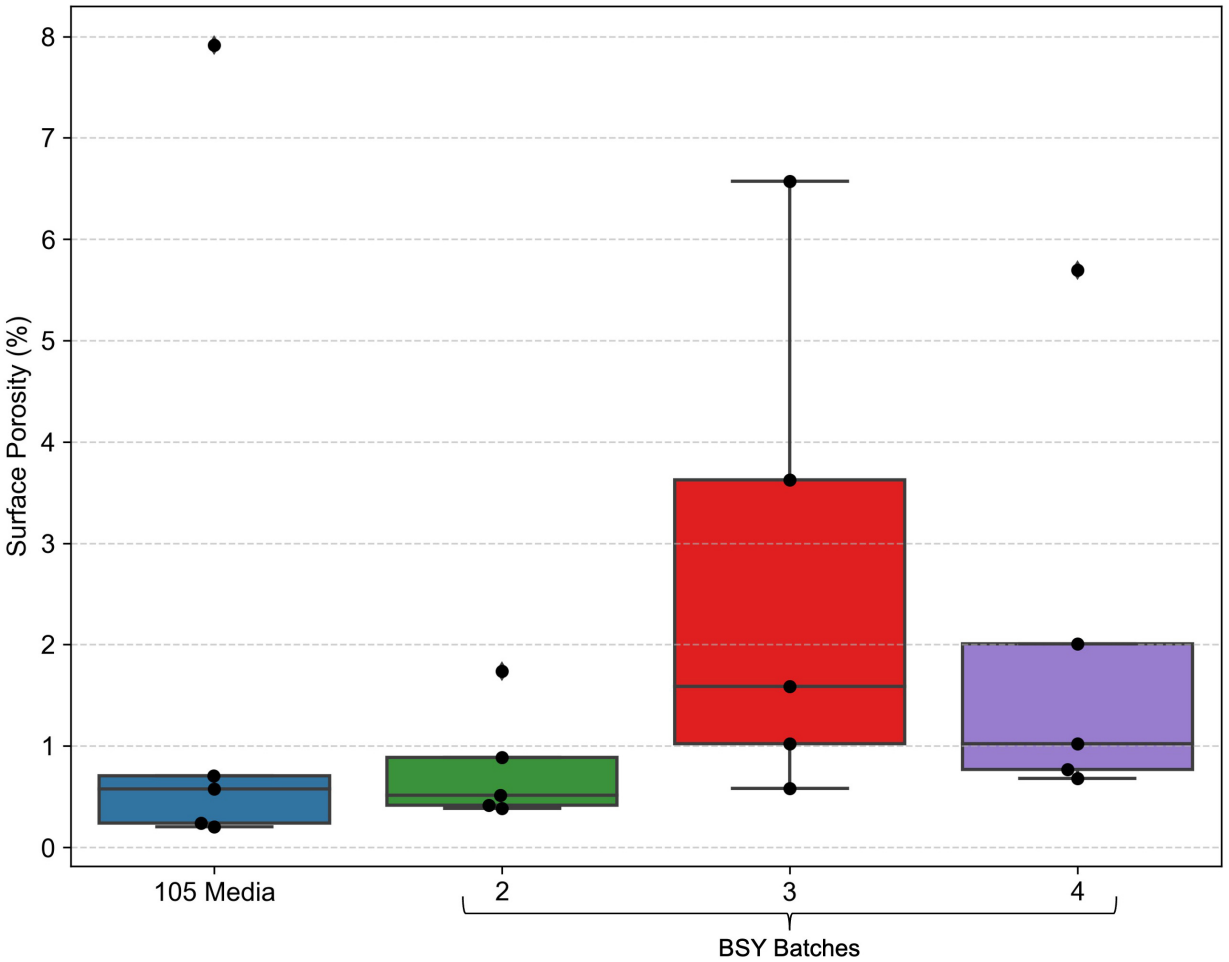
Percent porosity of cellulose pellicles grown in various growth media, as measured by analysis of SEM images (*n* = 5). Dots represent individual data points, ♦ points represent outliers. A Kruskal–Wallis test detected no significant differences between any groups (*p* = 0.1830).

SEM images of cellulose pellicles grown in 105 medium and in various BSY batches showed widespread and uniformly distributed pores across all treatment groups (Figure 3). Some debris was also visible in most images. Quantification of porosity (Figure 4) showed no obvious differences between any treatment groups, and no statistically significant differences were detected. Extreme outliers were present in both the 105 media and BSY batch 4 treatment groups.

**Figure 5:**
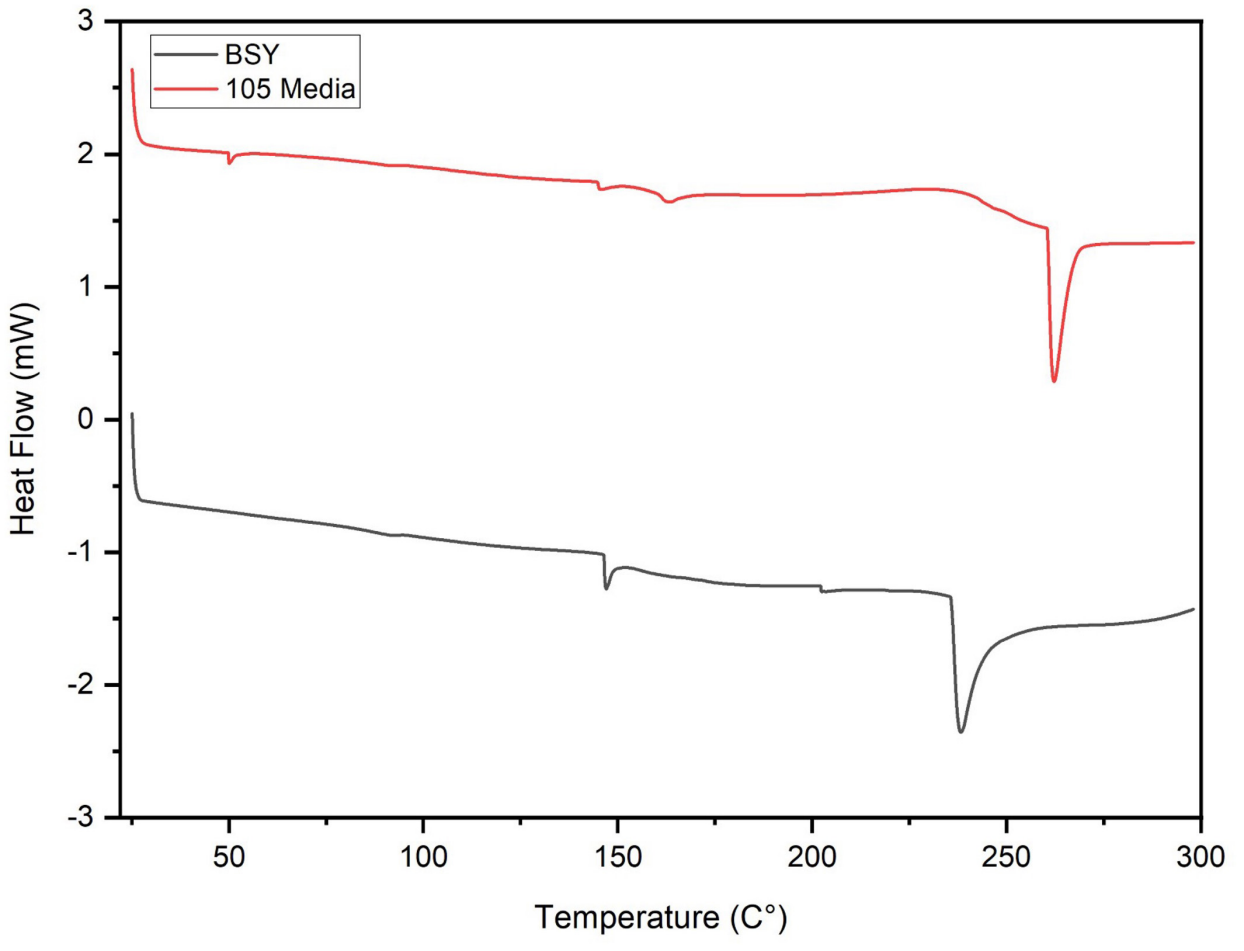
DSC thermogram of bacterial cellulose pellicles grown in BSY and 105 Media (*n* = 3).

In DSC analysis, both groups illustrated an endothermic peak at 145.31 °C for 105 media pellicles and 146.88 °C for BSY pellicles, attributed to residual water loss in the matrix (Figure 5). BC grown in 105 media exhibited additional peaks at 50.05 °C and 162.90 °C. Further endothermic peaks were observed at 238.08 °C and 262.17 °C for BC cultivated in BSY and 105 media respectively.

**Figure 6:**
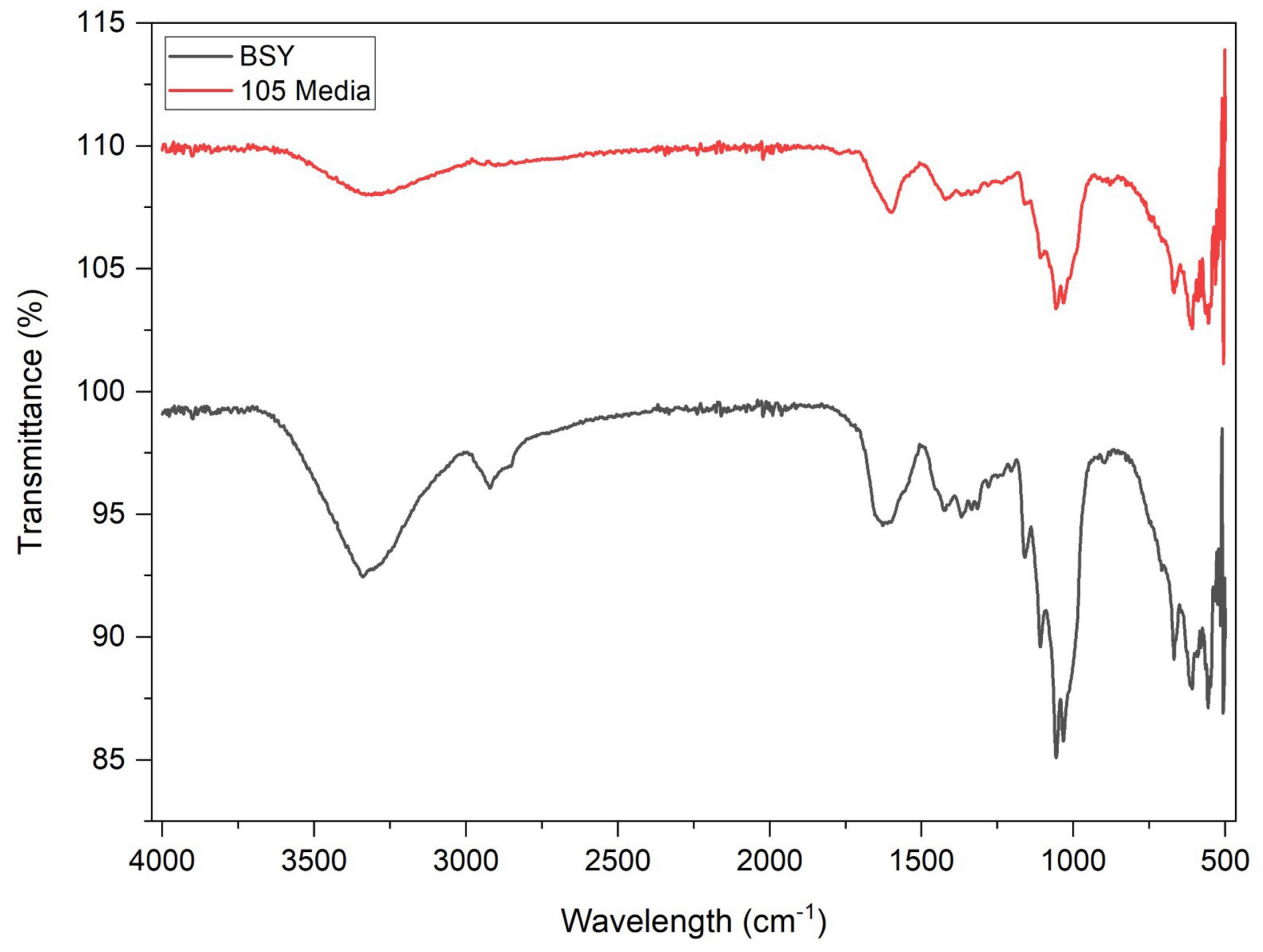
FTIR spectra of bacterial cellulose pellicles cultivated in BSY and 105 Media (*n* = 3).

FTIR spectroscopy produced almost identical spectra from pellicles grown in both 105 media and BSY (Figure 6). A broad peak was present in both groups at wavenumber 3,341 cm^−1^ and 3,314 cm^−1^ illustrating the stretching vibration absorption peak of –OH (hydroxyl groups). Both groups also showed an absorption peak corresponding to symmetric CH_2_ bending vibrations or O–H in plane bending (1,425 cm^−1^,) and another peak indicating C–O–C antisymmetric vibrations (1,161 cm^−1^). The main difference between the two groups was a peak at around 2,921 cm^−1^ corresponding to C–H stretching of CH_2_ and CH_3_ groups which was only seen in BSY-grown cellulose.

**Figure 7:**
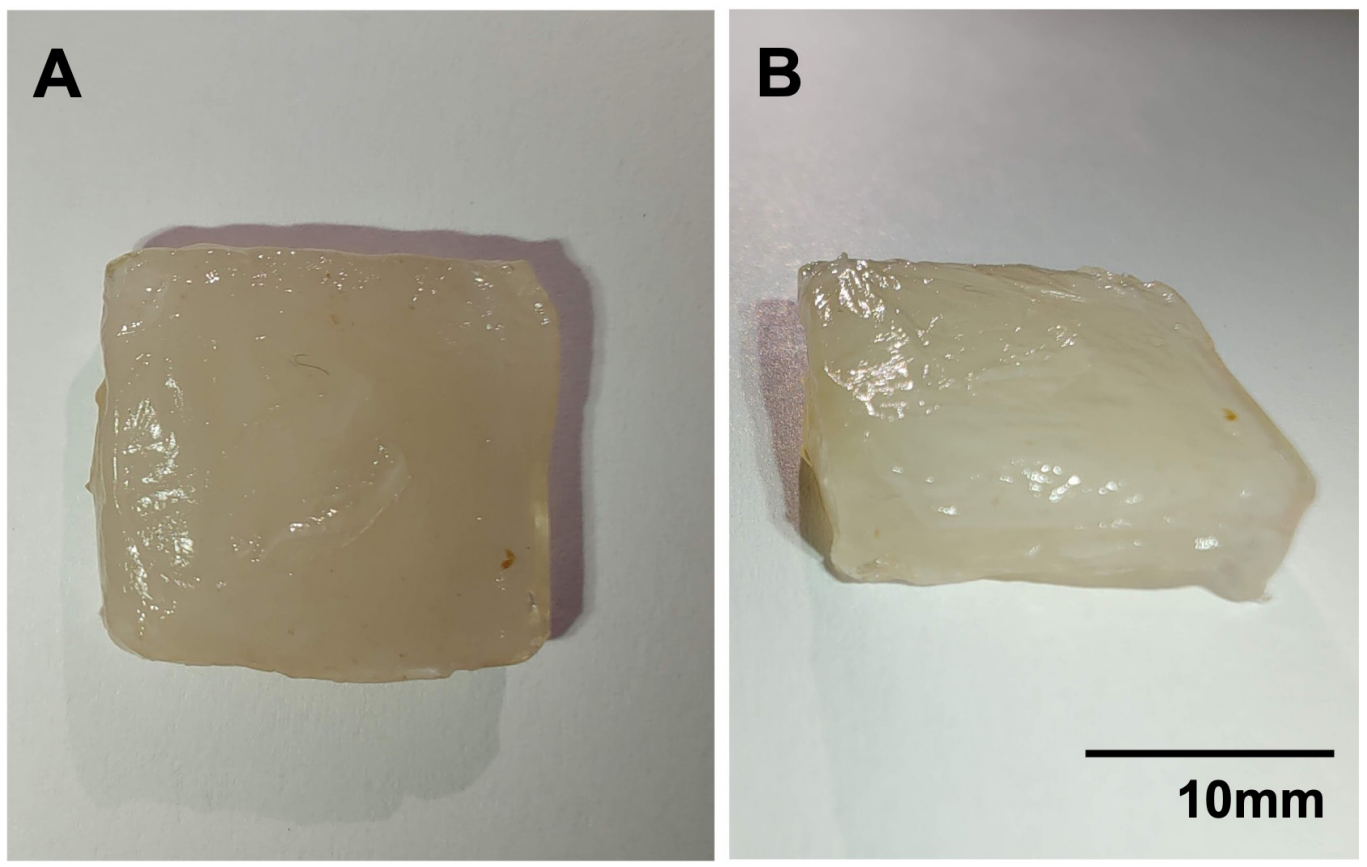
Photographs showing stacked cellulose-gel composites with a ruler for scale. **(A)** Composite photographed from above, **(B)** composite photographed from the side.

**Figure 8:**
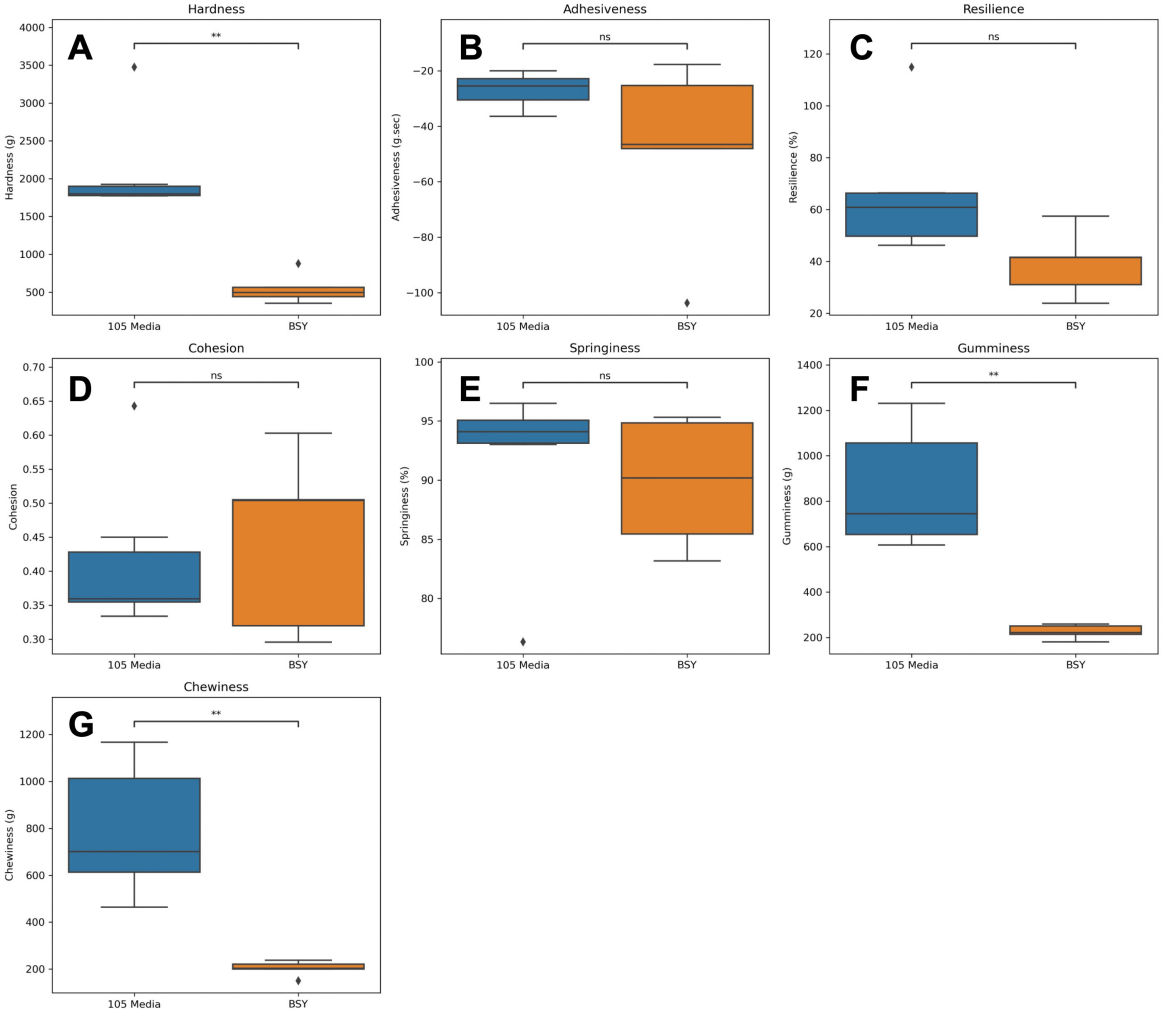
Various textural properties derived from TPA of cellulose constructs grown from either 105 media or BSY (*n* = 5). Plots show values for **(A)** hardness, **(B)** adhesiveness, **(C)** resilience, **(D)** cohesion, **(E)** springiness, **(F)** gumminess, **(G)** chewiness. ♦ points represent outliers. Welch's *t* test was used to assess statistical significance between treatments. Asterisks indicate significance level: **p < 0.01.

TPA was conducted on cellulose-gel composites (Figures 7, 8). Texture analysis showed cellulose-gel composites derived from 105 media and BSY exhibiting many similar textural properties, with no significant differences in adhesiveness, resilience, cohesion or springiness between groups (Figures 8B–E). BC grown in 105 media was however significantly higher in hardness (Figure 8A), and significantly higher in gumminess and chewiness (Figures 8F, G).

**Figure 9:**
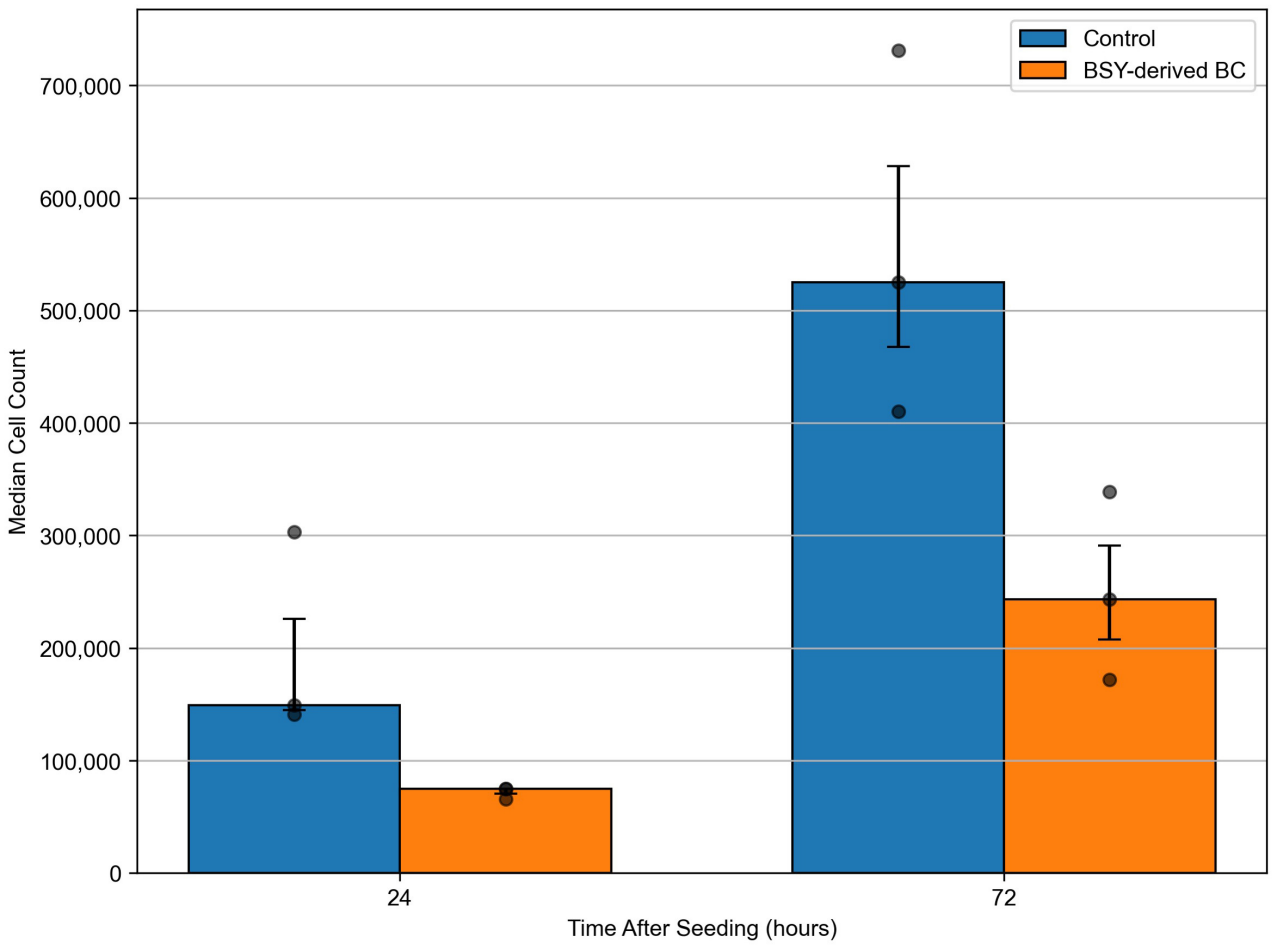
Mean cell count of L929 fibroblasts seeded on tissue culture plastic (Control) or BSY-derived BC, and counted at 24 and 72 h (*n* = 3). Median cell count is plotted with error bars representing the interquartile range. All other data points are shown as faded dots. Statistical comparisons were made using the Mann-Whitney *U* test. No significant differences between groups were detected at 24 h (*p* = 0.0765) or 72 h (*p* = 0.1000).

**Figure 10:**
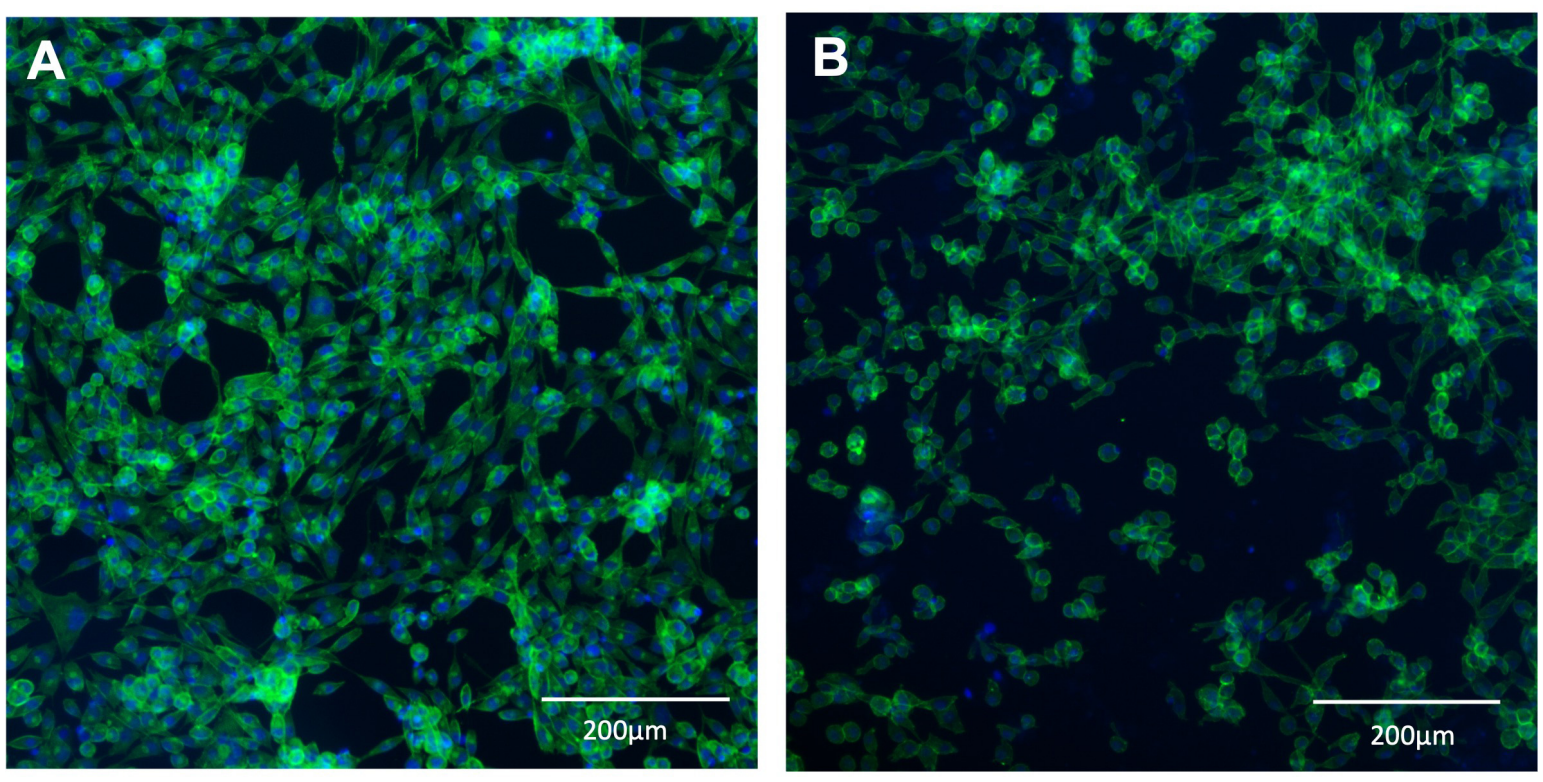
L929 mouse fibroblasts stained with DAPI (blue) and phalloidin (green). Cells were grown on standard 12-well tissue culture plates **(A)** or BSY-derived BC **(B)** for 72 h before being fixed and stained.

L929 fibroblast cells were seeded onto 12-well tissue culture treated plastic plates (control) or BSY-derived BC. Quantification after 24 h incubation showed no significant differences in cell attachment between the different substrates (Figure 9). After 72 h, the mean cell count had increased in the control group and on cellulose. Statistical tests showed there were significantly more cells present in the control group after 72 h than on cellulose pellicles. These results were confirmed by fluorescence microscopy, which showed visible differences in the quantity of L929 cells on tissue-culture plastic and BC after 72 h (Figure 10).

## Discussion

Achieving a high enough yield of BC from a given feedstock is key to its viability for CM applications, and yield is likely to be affected by feedstock composition. Metabolic analysis of different batches of brewing waste (Figure 1) demonstrated that substantial inter-batch variation exists in key compounds. This is likely to have arisen due to the different batches of BSY being sourced directly from an active brewing process. The bulk ingredients used in the fermentation process are dependent on the brewer's production schedule at the time of collection and therefore might vary from batch to batch. This aligns with previous findings of variability in mineral and nutrient content in BSY and other brewing wastes (29, 36). Beer itself can also vary substantially in its nutrient content (37), and presumably this would be reflected in any downstream waste products such as BSY. Given that significant differences in BC yield between batches were also observed (Figure 2), and strong negative correlations were found between yield and ammonium and potassium content, it is likely that variability in the composition of BSY must be considered in any commercial applications of BSY, such as production of cellulose pellicles for CM. However, the structural composition of conventional meat is heterogeneous and slight variations in the composition and yield of BC pellicles within acceptable boundaries may add to the appearance of a more natural product. To address this, further characterization of the chemistry of BSY may be advisable to ensure BC pellicles meet acceptable specifications. This might include recording when during the fermentation process yeast is collected, the age of the yeast, and the ingredients of the beer being produced, as all of these can influence the metabolic composition of BSY (29). The sugar profile of BSY could also be investigated further, as the current study only measured glucose, and BSY is likely to differ from pure glucose-based 105 media. It is possible that BSY from some production processes might be more suitable for BC manufacture than others. In the current study, no visible differences in microstructure were observed between pellicles grown in different batches of BSY or in 105 media (Figure 3). However, previous work has found differences in BC crystallinity, as measured by x-ray diffraction, depending on growth media composition (38), which should be explored in future studies of BSY-derived BC.

The apparently negative effects of ammonium and potassium in BSY could be attributed to them raising the osmolarity or ionic strength of the medium to high levels, which has been shown elsewhere to inhibit bacterial growth and cellulose synthesis in particular (39, 40). Other nutrients such as magnesium, sulfur, and phosphorus as well as certain vitamins are also known to influence BC production (23, 41), therefore future studies could investigate levels of these compounds in BSY. Notably, BC yield in BSY batches 3 and 4 equalled that in 105 medium (Figure 2), showing that *Komagataeibacter xylinus* can grow well in at least some types of brewing waste, strengthening the case that BSY could be a viable feedstock for large scale BC production for CM. To address variation in compounds that influence BC yield, further characterization of the chemistry of BSY may be advisable to ensure BC pellicles meet acceptable specifications for CM. Future studies could also investigate any differences in structure or biocompatibility between BC pellicles derived from different batches of BSY.

High porosity is key to the suitability any scaffold for CM production, as it mimics the natural extracellular matrix (ECM), allowing cells to attach to and penetrate the scaffold and efficiently exchange nutrients and waste products (42). Variability in BSY composition did not appear to influence surface porosity, which was similar across BC grown in all BSY batches and 105 medium (Figure 4), indicating that the structure of BC is not affected by variability in BSY. Surface porosity was similar to values reported elsewhere for BC (43). However, the SEM technique used here only allows measurement of surface porosity, whereas many studies report porosity values that include the internal material structure, and which are much higher than the surface porosity found in this study (44, 45). Future investigations into the porosity of BC from brewing waste should make use of methods such as micro-CT or mercury porosimetry to more accurately assess porosity, including in the internal structure of BC (45). If porosity of BSY-derived BSY is lower than required, there are several techniques that could be used to increase it. It has been reported that decreased cultivation time and inoculation volume can increase porosity, and that porosity may actually be inversely correlated with overall cellulose yield (44). Alkali treatments of BC have also been found to raise porosity, and although NaOH washing was utilized in this study, other alkaline treatments have been found to increase porosity more (44). The exact carbon source in the growth medium may also influence porosity (46), which may warrant measurement of alternative carbon sources like sucrose, maltose, fructose and mannitol in future metabolic analysis of brewing wastes. Another technique that may be useful is the development of macroporous BC by growing bacteria in a foaming growth medium (47). The viscous nature of BSY could lend itself well to this application, and the foaming structure could increase the surface area available for cell growth and aid in producing 3D CM products.

In FTIR analysis, both BSY and 105 medium-derived BC produced broad peaks at 3,341 cm^−1^ and 3,314 cm^−1^ characteristic of BC (Figure 6) (48, 49). However, only the BSY samples produced a peak at 2,921 cm^−1^, which suggests the presence of organic impurities in the sample (50). This is supported by DSC analysis, where BC from 105 media and BSY exhibited endothermic peaks at 238.08 °C and 262.17 °C respectively, likely corresponding to the melting of the crystalline phase (Tm) of BC (Figure 5) (51). The lower temperature of the peak in the BSY group could indicate a higher level of impurities in these samples (51). This could have negative implications for CM applications as high purity is one of the main advantages of BC over other scaffolds such as plant cellulose (52). The presence of impurities in BC could also have implications for food safety and cell growth on cellulose. It is possible washing BC pellicles in hot NaOH for an increased duration or using a higher concentration of NaOH may achieve a higher level of purity. However, it is also possible that the high protein and vitamin content of BSY (32) could mean that any impurities are nutritionally beneficial for consumers of CM products. More aggressive washing could also alter properties such as porosity and mechanical strength which may be relevant for CM applications (53), so should be tested in future studies.

For CM to gain consumer acceptance, it should aim to match the textural and mechanical properties of conventional meat products. TPA performed on cellulose-gel composites (Figure 7) showed no differences between BSY and 105 media-derived BC in adhesiveness, resilience, cohesion or springiness (Figures 8B–E). Cohesion values were found to be lower than those reported for some conventional meat products (54). 105 media and BSY-derived cellulose gave cohesion values of 0.42 and 0.47 respectively, while values of above 0.8 are reported for both sausage and turkey. Cohesion is a measure of the strength of internal bonding (55), so a low value may indicate that the Vege-Gel used in this study to bind cellulose sheets together (Figure 1) is not strong enough for CM purposes, and that alternatives need to be explored in future. A wide range of plant-based glues are under development; these may be suitable adhesive agents for BC-based CM products (56). Springiness values of both BSY and 105 media-derived cellulose were however both similar to conventional meat values at 91.5% and 89.8% respectively compared with 80%−85% for sausage and turkey (54). There were significant differences between groups in hardness but notably values for BSY-derived cellulose at 546.6 g (~5N), are much closer than 105 media-derived cellulose at 2,092 g (~21 N) to sausage and turkey at around 7 and 5 N respectively, with a similar pattern seen in chewiness (54). This could suggest that BC derived from conventional medium is too chewy for CM and that BC from BSY is better suited. The reduced hardness of BSY-derived BC could be partly explained by the impurities detected by DSC and FTIR (Figures 5, 6), as purification using alkaline treatment has previously been linked to mechanical strengthening of BC (57). Other mechanical properties of BC such as tensile strength may also be relevant to CM applications (58), so these parameters should also be measured in future studies of BSY-derived BC. Furthermore, there is evidence that different strains of bacteria produce BC with different mechanical properties (58). Only a single strain of *K. xylinus* was used in this study, so future studies could attempt production of BC using other strains and investigate any changes in BC properties relevant to CM. Edible binders other than Vege-Gel, such as alginate or agar, could also be used to create BC composites. These may produce different mechanical properties more or less amenable to CM products, and this could be tested in future experiments.

Another crucial quality for any potential CM scaffold is the ability to facilitate cell attachment and proliferation under xeno-free conditions. The current study demonstrates that the serum-free medium used is compatible with cell attachment and growth. At 24 h after seeding, L929 cells attached to BSY-derived BC as well as in the control tissue culture treated plastic group (Figure 9). At 72 h, significantly more cells were quantified on control tissue culture treated plastic compared with BC. However, microscopy did show cells present on both substrates after 72 h (Figure 10). Is it important to note that due to the single batch of L929 cells used in this experiment, batch effects cannot be ruled out, and future studies should aim to test multiple batches. Tissue culture treated plastic consists of exposing a polystyrene tissue culture vessel to a plasma gas and has been optimized to result in the plastic surface becoming more hydrophilic. Oxygen plasma treatment has previously been employed to improve hydrophilicity, morphology and ionic strength of various cell substrates, thereby increasing biocompatibility (59). Several types of plasma treatment exist, including O_2_ but also N_2_ and CF_4_ (60). It has been reported that CF_4_ was most effective at promoting cell attachment and proliferation (60), and this approach could be further explored in future to improve the surface properties of BC for cell attachment. Another potential strategy is treatment of the cellulose surface with Arginyl-Glycyl-Aspartic Acid (RGD) peptide-containing proteins like vitronectin and fibronectin, which has been shown to improve cell proliferation on other forms of cellulose (61), and could be applied to BC in future. It is also possible that more aggressive NaOH washing of BC pellicles, as discussed above, could enhance biocompatibility by increasing the purity of BSY-derived BC.

The L929 fibroblast cells used in the current study are a highly consistent cell line widely used for testing biocompatibility and cytotoxicity of novel substrates (62, 63). While fibroblasts do not yield muscle or fat, up to 10% of conventional meat is composed of connective tissue including fibroblasts and these play a key support role in development of muscle fibers (64), therefore they are relevant to development of CM products. Furthermore, CM is intended not only for human consumption but also for obligate carnivore domestic animals like cats (65); murine cells such as L929s may be suitable for this purpose. Given that the current study has demonstrated proof-of-concept for cell culture on BSY-derived BC, future studies should test other cell types and species, to enhance the relevance of this scaffold material for CM applications. Additionally, co-cultures of multiple cell types are believed to be necessary to reduce costs and improve organoleptic properties of CM (66). A longer-term goal of future work should be to develop co-culture or layering techniques using BSY-derived BC to enable realistic, 3D CM products. Whether BC can support such processes will likely depend largely on its properties of porosity, biocompatibility and mechanical properties that are explored in the current study, therefore future work should investigate these more thoroughly.

In the current study, small quantities of BSY were collected and BSY composition varied depending on the brewer's output at that time. Scaling up the BSY to BC to CM production process would likely require greater coordination between brewers and BSY-users regarding their ingredients and production schedules. This would ensure consistency between batches, helping mitigate any problems arising from the variable composition of BSY feedstock, as encountered here. Consistency is likely to be key in any potential food-grade CM product. The current study also only provides data on 8 common metabolites, but other metabolites could influence BC yield and structure. A broader metabolomic analysis is therefore advisable in future, which would increase understanding of challenges which may be faced in scaling this process. Additionally, only static BC cultures were used, which may provide insufficient yield for large scale CM production at low cost (67). Alternative culture methods such as stirred-tank bioreactors or continuous cultures should be tested for suitability in CM production. This will help understand how BSY-derived BC could be mass-produced. Finally, no methods of enhancing biocompatibility of BC were tested in the current study. Based on the results described here, some improvement in this area will be needed if BC is to be used to manufacture CM at scale. These methods will also need to be cost-effective to make CM competitive. Increasing biocompatibility should be a high priority for future work.

**Figure 11:**
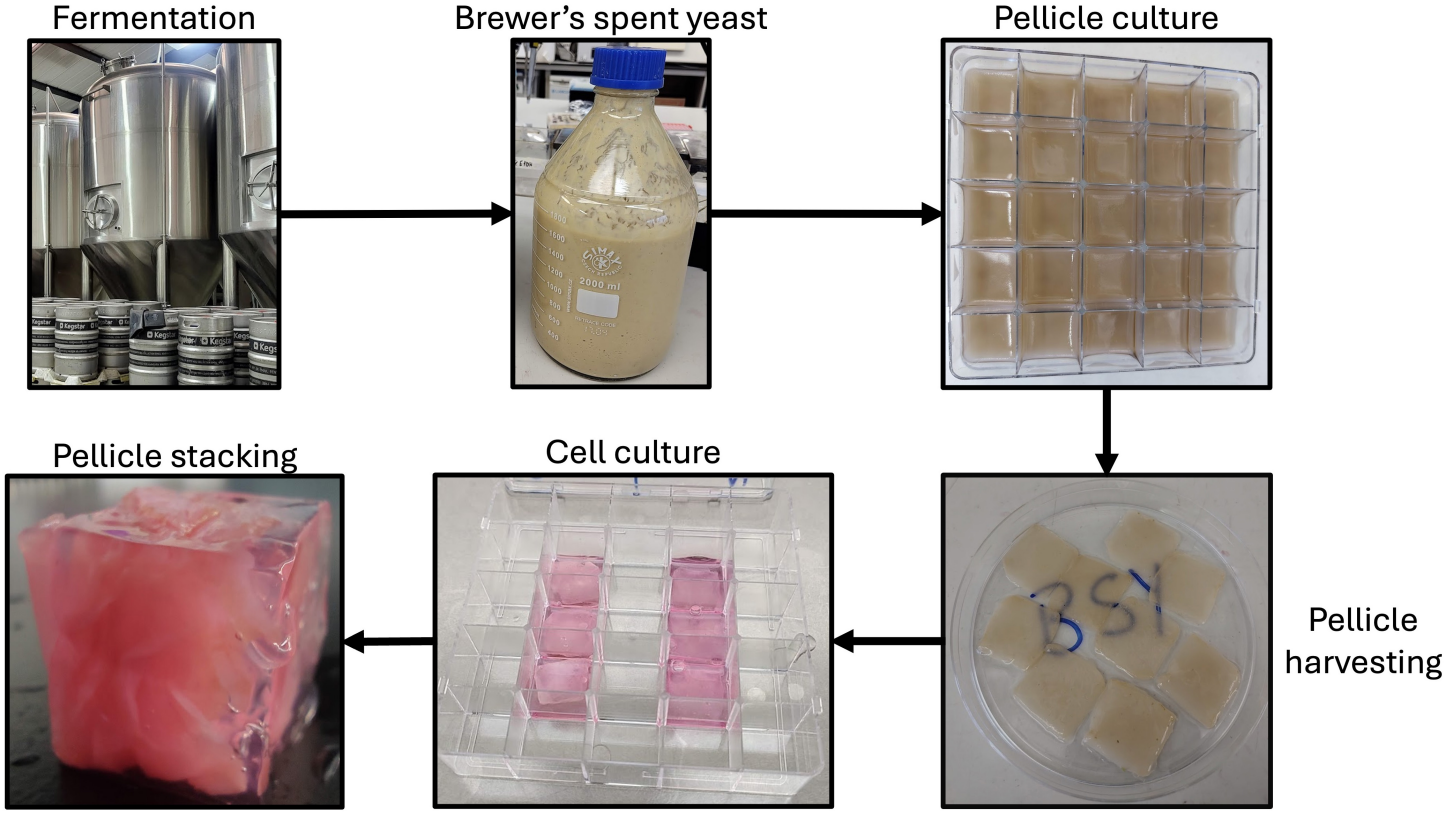
Flowchart showing the production process proposed in the current study. BSY is taken from the fermentation tank and used to culture *K. xylinus* bacteria to produce cellulose pellicles. Pellicles are then harvested, seeded with cells, then stacked and encased in gel to create a cube.

In conclusion, the current study demonstrates proof-of-concept for a production process using BSY-derived BC pellicles in a CM product (Figure 11). Further studies will be necessary to optimize this production process and demonstrate how different cell types that contribute to muscle and fat content of meat interact with the materials and whether the materials can be used to create multifaceted constructs beyond cubes. The use of BC pellicles derived from brewing waste could provide a sustainable and cost-effective approach to CM and warrants further exploration.

## Data Availability

The raw data supporting the conclusions of this article will be made available by the authors, without undue reservation.
